# Problems and Implications of Shelter Planning Focusing on Habitability: A Case Study of a Temporary Disaster Shelter after the Pohang Earthquake in South Korea

**DOI:** 10.3390/ijerph18062868

**Published:** 2021-03-11

**Authors:** Mikyung Kim, Kyeonghee Kim, Eunjeong Kim

**Affiliations:** 1 Department of Housing and Interior Design, Chungbuk National University, 1 Chungdaero, Cheongju, Chungbuk 28644, Korea; mkmkkim@chungbuk.ac.kr (M.K.); rlarudgml721@naver.com (K.K.); 2 Dodam Design and Research, #101-904, Hangangdaero 211, Yongsan-gu, Seoul 14322, Korea

**Keywords:** temporary disaster shelter, habitability, shelter planning

## Abstract

Habitability is an essential concept for shelter planning in terms of supporting victims’ right to life with dignity and recovering from what they suffered. The study aimed to identify problems and needs in shelter spaces and suggest measures to improve shelter space plans by conducting a case study in South Korea. The temporary disaster shelter in Pohang built right after the earthquake (2018) was selected as a case subject. From the literature review, a framework consisting of four concepts of habitability (safety, health, sociality, comfort) and four shelter zones (entry, residential, service, special needs zone) was developed for the in-depth interviews and analysis. The field study and in-depth interviews with victims, staff, and volunteers were conducted to collect problems and needs regarding shelter space planning. The results showed that the entry zone needed improvements in ‘protection’, ‘prevention’, ‘sanitation’, ‘accessibility’, ‘area’, and ‘privacy’. The residential zone lacked ‘area’, ‘privacy’, and ‘indoor environmental quality’. The service zone problems were mainly seen in the categories of ‘area’ and ‘privacy’. The special needs zone was less habitable in the categories of ‘protection’ and ‘area’. To appropriately respond to victims’ urgent needs, the temporary shelter planning should secure enough space beyond the legal minimum standards, provide sanitation and indoor environmental quality management, and separate spaces by function and user type.

## 1. Introduction

A 5.4 magnitude earthquake struck Pohang, South Korea in November 2017, which led to large-scale destruction with nearly 1300 people left homeless in the wake of the disaster. The evacuation was prolonged and people were housed in a temporary disaster shelter prepared hastily in a gymnasium. Due to the frequent aftershocks, residents had to stay in the temporary shelter for over two years, suffering in unhealthy conditions both in the cold winter and the extreme heat of the summer. This led to physical illness and severe psychological anxiety. Tents were provided for survivors to protect their privacy, but this led to various problems such as the tents being too narrow for people to live in and they did not block external noise.

A temporary shelter should be safe from external potential physical risk factors and provide stability for those living there to regain their physical health and mental security [1,2,3]. Prior studies emphasized that the concept of habitability should be applied to temporary disaster shelters [4,5,6]. Habitability means suitability as a residence, and suitability implies the provision of a shelter, heating system, sanitary condition to prevent infection, indoor quality (noise, smoke, drugs, etc.), and the drinking water supply. It also includes the broader aspects such as a close association with local communities, people’s will to recover, and consideration of various needs based on individuals’ physical and emotional states [6]. Therefore, as an essential concept for building and managing a shelter to accommodate those who survived a disaster, habitability should not be seen as a minimum standard for survival but be viewed as a building a soft environment that can be respected and dignified as a human. In this aspect, to better support the concept of habitability, temporary shelter for disaster survivors should provide a physically and psychologically stable living environment that considers health, safety, hygiene, and a prolonged recovery stage in various disaster situations.

The Ministry of the Interior and Safety in South Korea issued formal guidelines [7] for supporting survivors in temporary disaster shelters during the early stage (within 24 h), emergency stage (3–5 days), and recovery stage (more than 5 days), depending on the period. In the early stage, the guidelines include indoor and external facility inspections, guidance on initiating shelter operations, and preparing plans for prolonged evacuation. The emergency stage involves the preparation of (a) registration forms for disaster survivors and (b) spatial zoning and arrangement according to function and needs within the shelter environment. In the recovery stage, for better response to prolonged evacuation, the required spaces and facilities are checked and service types and qualities expanded.

Recently, temporary shelters have seen many improvements in their operation and management. However, specific details regarding shelter planning for a stable living environment considering the physical and psychological health of disaster survivors have not been fully examined and reflected in the guidelines issued in South Korea.

A temporary shelter houses people for the short term after a disaster. To provide survivors better service and a decent quality of life, it is important to build a physically and psychologically stable living environment. For this, one needs to view the situation on-site and understand survivors’ direct and indirect needs. Against this background, this study aims to identify problems and actual needs for each space within the shelter and suggest potential measures to improve the shelter space plan for habitability. For the field research, data were collected from the H indoor gymnasium in Pohang, where the largest number of survivors were temporarily housed after the 2017 earthquake.

## 2. Literature Review

### 2.1. Habitability in Temporary Disaster Shelters

Habitability is an essential concept for establishing and managing temporary shelters. It ensures they can live safely over the long term. It is a humanitarian response toward creating an environment that considers the quality of life, health, welfare, and recovery from the crisis [3,8]. People can live a healthy life and prepare for a future disaster only by securing a safe and appropriate living space [8]. To this end, reflecting on survivors’ opinions regarding a safe environment, comfort, adaptability, sanitation, community connections, privacy, and functionality could help build an environment that increases a person’s will to recover [3,4,9,10,11].

The concept of temporary shelters’ habitability can be confirmed through prior studies. Um et al. [12] suggested protection, privacy, safety, psychological stability, health services in the form of medical support, and hygiene and pollution management as crucial factors affecting habitability. The American Red Cross [13] mentions safety, cleanliness/hygiene, consideration toward vulnerable people, diversity, and privacy, while Bashawri et al. [5] explains it through environmental aspects (external environment response, safety, hygiene) and sociocultural aspects (cultural differences, security, and communication). Sphere [8] suggests each family affected by the disaster needs to be provided with adequate space for basic living, and that local culture and lifestyle should be considered to accommodate the diverse needs of family members for sleeping, food preparation, and meals. The Sphere handbook also explains that physical security, privacy, protection from the weather, optimal lighting conditions, ventilation, heat, and comfort should be addressed to achieve adequacy for an affordable, habitable, culturally acceptable, and accessible and usable shelter environment. Moreover, the residential areas should be safe for cooking, toileting, laundering, bathing, living, socializing, and recreation, ensuring victims’ privacy.

Choi, Kim, and Kim [6] developed a shelter planning guide for temporary shelters in South Korea. The authors classified the concept of habitability into four aspects to build a research framework, and based on the analytic framework, 46 shelter planning guidelines were developed after conducting literature reviews and interviews. They suggested a framework consisting of 4 main categories (safety, health, sociality, and comfort) and 10 sub-categories (protection, prevention, sanitation, medical support, vulnerable people, accessibility, community, area, privacy, and indoor quality) to conduct interviews and develop guidelines. In this study, we adopted the framework as a part of an analysis method and explanation of the concept of habitability as shown in Table 1. The category of main and sub-concepts was borrowed, but the slight explanations were added or modified in the description part. The adopted framework (category of habitability) was combined with the shelter zones which were classified by the authors, and the analysis matrix with the x and y-axis was introduced in the analysis section.

Table 1 introduces the critical concepts of habitability which play important roles to build a suitable shelter as a residence. The concept of “safety” involves protection from potentially dangerous situations such as additional disasters, bad weather conditions, and trespassers, etc., and the provision of secure storage areas. “Health” includes hygiene and medical assistance facilities and services for survivors’ physical and psychological health. “Sociality” refers to the connectivity of the temporary shelter to neighborhood facilities, the absence of pedestrian obstacles, and its location and accessibility. “Comfort” refers to personal space for privacy, minimum living area, natural/artificial lighting, heating, ventilation, and other indoor environmental factors related to survivors’ living space.

### 2.2. Zoning for Temporary Disaster Shelters Focusing on Habitability

A temporary disaster shelter requires various functional space types, such as a residential space for daily living, a public area for medical treatment and administrative services, meals, and communication, and a dedicated employee space to accommodate diverse users.

Regarding shelter zoning, The Department of Justice (DOJ) [14] included entrance spaces (boarding/unloading, parking lots, sidewalks, entrances, corridors), living spaces (sleeping areas, toilets, showers, public telephones, drinking fountains, dining areas), and others (personal/family toilets, medical care room, temporary toilets). FEMA [15] classified shelter spaces like a parking lot, space for pick-up and drop-off, waiting, registration, residence, children and family, pet, snack bar, dining, medical care, resting, staff, management, and warehouse based on accessibility and functional support. The US Centers for Disease Control and Prevention (CDC) [16] proposed a separate isolation area in case of an outbreak of infectious disease by emphasizing the maintenance of a sanitary environment for sustainable living in the temporary shelter. Of particular importance is ensuring hygiene in public areas such as entrances, dining spaces, medical care spaces, pet areas, and children’s playrooms to prevent the spread of infection [17,18].

Based on the functional spaces mentioned above, the spaces in a temporary shelter can be divided into four categories: entry zone, residential zone, service zone, and special needs zone. The entry zone includes parking spaces, entrances, registration/waiting spaces, and it should be accessible by emergency vehicles and survivors’ vehicles. People who are unable to walk or have disabilities should be able to easily access these spaces. Besides, since the entry zone is marked by the constant movement of people in and out, thorough hygiene management should be planned in this zone. A hand washstand or hand disinfection system should be installed near the entrance, and a separate independent waiting area for people with infectious diseases should be provided. Along with the hygiene issue, plans must be made for protecting survivors’ safety and privacy. For this, an access security checkpoint should be provided right in front of the entrance to screen outsiders or reporters.

The residential zone includes the living space, toilet, shower room, laundry area, dining and meal-preparation area, and rest area. The appropriate area per person should be secured considering the composition of the various personnel, the number of survivors staying in the shelter, types of family/individuals, gender, and special needs for vulnerable people. The space layout for the residential zone should be planned to minimize noise from the outside, passages, or public areas such as lounge, dining room, playrooms, etc. Physical obstacles should be removed from passages to provide people a barrier-free environment. Besides, it is important to provide a pleasant and hygienic living environment by maintaining a proper temperature, humidity, and lighting. Hand washstands or hand sanitizers should be placed at regular intervals.

The service zone covers the management space of employees and medical/isolation spaces. An adequate area for employees and managers should be secured for both work and rest. Separate entrances and paths for staff should also be considered. Medical care and isolation spaces, accessibility, hygiene, and privacy should be considered.

The special needs zone includes rooms for children, women (pregnant/nursing mothers), and pets. For convenience, rooms for children and pregnant/nursing women should be adjacent to the toilet and hand washstand. These spaces should also ensure all practices involving safety, hygiene, and privacy by removing barriers, managing hygienic cleaning systems, and providing partitions. The space for pets should be planned in isolated or separate spaces away from the residential zone considering the dimensions, distance, drainage facilities, and the species and size of the pets.

Table 2 shows the classified hierarchy of temporary shelter zones by their functions. The shelter zones are divided into four different areas, and each zone has a public or private character depending on the function it performs involving various stakeholders such as victims, managers, and volunteers.

### 2.3. Research Method

The research questions for this study are: (1) How is a temporary shelter space in South Korea organized for vulnerable people right after a disaster occurs?; (2) What are the main problems identified for each zone in the temporary shelter regarding habitability?; (3) What can be improved to better support the shelter space planning?

For this study, qualitative research methods were applied. We selected a temporary shelter located in Pohang for the study and visited the place to conduct in-depth interviews with people who lived through the disaster. The interviews were conducted twice: (1) the first interview focused on the identification of people’s overall needs, and the shelter’s physical environment and atmosphere; (2) the second interview collected more voices from the field, specifically from vulnerable people. The interviews were based on the semi-structured questionnaire which was developed from the literature review.

This study examines a temporary shelter, an indoor gymnasium, in Pohang (South Korea) in the aftermath of the earthquake that occurred in 2017. The shelter accommodated 430 survivors at that time. In-depth interviews were conducted with survivors, volunteers, and staff who stayed and worked in the shelter. We also examined the original floor plan of the gymnasium to identify how the gym’s indoor space was organized as a shelter and how survivors responded to the indoor environment.

Data were mainly collected from the interviews due to the strict access restrictions at the shelter. The first interview was conducted on 27 January 2018, and the second interview was conducted on 27 March after the 4.6 magnitude aftershock on 11 February. Access to the inside of the shelter was strictly regulated for visitors, and accordingly, articles and broadcast materials were collected to analyze the inner space plans and environment.

In-depth interviews were conducted in the (a) first interview with 20 survivors, four volunteers, and two managing staff who stayed in the shelter for more than a month and (b) a second interview with 12 survivors, one volunteer, and one staff manager who had spent more than three months in the shelter. A total of 40 people participated in the in-depth interviews. The interviewees were discreetly approached by researchers and selected based on the period of shelter stay, user type (survivors/volunteers/staff), and their willingness to participate in the study. The interviewees were asked to informally describe their experience in the shelter based on the questionnaire’s framework, and the problematic issues regarding the environment’s space were discussed in-depth. Each interview was recorded with the permission of the interviewee, and after the interview, the data were transcribed and analyzed by finding concepts in meaningful words or phrases related to the research subject.

The questionnaire for the interview consisted of 30 questions (entry zone (9), residential zone (12), service zone (3), the special needs zone (6)) as shown in Table 3. The researchers asked people questions based on the questionnaire but allowed people to talk freely instead of following the format of the questionnaire. The questions did not proceed in order but varied according to the situation so that the flow of the interview was not disturbed.

## 3. Results

### 3.1. Floor Plan and Physical Environment of the Shelter

#### 3.1.1. Entry Zone

The parking area was used as a space for providing snacks, meal booths, laundry vehicles, etc. There was insufficient parking space for disaster survivors as staff vehicles and ambulance vehicles occupied the area. The main entrance was the only passage open for disaster survivors after the first earthquake, but with the aftershock that occurred, the emergency exit was opened in case of an emergency evacuation. The registration/waiting space was located between the main entrance and the lobby; it also functioned as the management space and as a mobile phone charging station.

#### 3.1.2. Residential Zone

In the living (sleeping) area on the 1st and 2nd floors, tents were set up for each person or a family. Sanitary space for toilets, showers, and changing rooms was planned on the first floor. Survivors could also use the facilities in the nearby town office and a bath ticket to use the public bath nearby. This showed that hygiene management was considered important in the planning and management system with diverse options for people. The laundry/drying space was not planned, but laundry vehicles provided laundry and drying services. Dining/meal-preparation space comprised meal vehicles and dining booths were planned in the parking space, but as the shelter stay continued, four dining spaces were later reduced to one. The dining area was used as a lounge for information communication and watching television except during mealtimes. There was no separate resting space planned inside the temporary shelter, and accordingly, the space between tents in the living area was used as an informal lounge.

#### 3.1.3. Service Zone

The management space was planned in the entrance lobby at the main entrance and was furnished with tables and chairs for registration and access confirmation. A separate resting area was not provided for staff and volunteers. The volunteers spent most of their time in booths located in parking spaces. Medical services were located in the pulpit area in the hall, which was divided into a treatment space and waiting area using partitions for privacy.

#### 3.1.4. Special Needs Zone

There was a separate designated space for children inside the shelter for their medical treatment and for psychological counseling for those who had been affected by the trauma of the earthquake. No separate room was allocated for infants and toddlers, or pregnant or nursing mothers; thus, inconveniencing them. There was also no planned pet area.

### 3.2. Problems Pertaining to Habitability

#### 3.2.1. Entry Zone

From the interviews and field study, it was identified that the parking lot was planned mainly for service spaces such as dining booths and parking laundry vehicles. There was no secure parking space for survivors and, therefore, poor access to the shelter. Furthermore, garbage was treated in the parking lot, causing an odor and public sanitation problem. The emergency exit was open only after the aftershock, but survivors were not aware of its location, thus causing bottlenecks at the main entrance initially. The door handles were easily contaminated by multiple users, resulting in problems such as safety and sanitation. The doubling up of the registration/waiting space as a management space and as a mobile phone charging station resulted in privacy issues such as leaking personal information due to insufficient space (Table 4).

This led us to understand that parking spaces must be planned to secure accessibility for survivors. The parking lot shared space with relief services. However, if not for everyone, it was necessary to provide parking spaces for older adults, the disabled, and families with children. A separate garbage disposal space plan was required to maintain cleanliness and hygiene. If the main entrance is the only access point, an emergency exit should be considered as a sub-entrance because of safety and hygiene. A hand washstand or hand sanitizing system should be provided at the entrance.

*“After dismantling the service booth, parking for disaster survivors was allowed in the parking space near the entrance. Before that, parking around the shelter was not allowed for survivors, and even older adults had to walk up outside the shelter, which made it difficult for them.”* (survivor, 60s, woman).

*“Since many people use the same entrance door every day, it becomes dirty and sticky. So, sometimes I wipe it with a wet tissue to clean the handles and door surface.”* (survivor, 70s, woman).

*“Even when there was an emergency exit open to the public after the aftershocks, people still flocked to the main entrance door making it more dangerous.”* (survivor, 40s, man).

#### 3.2.2. Residential Zone

In the living space, the survivors were provided a tent for two persons, and storing personal items securely in the tent became difficult; size options were not considered for a family unit (more than three persons). Family members had to live apart during the shelter stay and store personal items in public lockers located far away from the residential zone. Some survivors continued to go back to their damaged houses to bring personal necessities left in the house. These experiences brought up issues of privacy and protection. Besides, the passage between tents was narrow, making it difficult for people to pass through. People did not sleep well at night due to the noises, and these experiences were related to the sustainable aspects of accessibility and indoor quality.

Those who lived on the 2nd floor had poor accessibility to the toilets and the mobile phone charging stations that were on the 1st floor. Older adults were reluctant to go toward toilets or shower rooms if the spaces were far from their living area. The careless arrangement of sanitary spaces without considering sex, such as having a male toilet next to women’s shower rooms or a women’s toilet next to male shower rooms made people uncomfortable whenever they used these spaces. These revealed the need for the rearrangement of spaces for accessibility (Table 5).

There was no drying space planned for laundry. Personal laundry when dried outside the tent was exposed to others, and when dried inside, increased the humidity causing an odor. Women especially had difficulty finding proper drying space for their underwear. This situation was highly related to aspects of privacy and sanitation.

Since the dining/meal-preparation space was in the parking lot outside the shelter, people had poor access, especially when it rained or snowed, and they became exposed to the cold. The rest area had privacy problems, such as noises and conversations overheard through the empty spaces between tents, which were used as a temporary lounge. From this, it was understood that in the living space, tents of various sizes and types should be provided for storing personal items and protecting family units. A storage plan such as prefabricated storage furniture is needed to safely store personal items (Table 6).

From the identifications mentioned above, it can be interpreted that the sanitary space arrangement plan should consider sex and accessibility, particularly for older adults, by planning the residential zone near sanitary spaces. Besides, the dining area should preferably be located inside the shelter, and if it is difficult to secure an indoor space, it is necessary to consider installing a refrigerator to keep people’s food fresh. A place to relax must be mandatorily provided for people to converse and to ensure their emotional wellbeing. The scale and size of the lounge may be small considering the need to protect personal or family unit privacy.

*“The tent size is so small that it is uncomfortable to change clothes inside. My husband stays in a relative’s house and I stay alone here for convenience. I don’t have enough space to keep my stuff, and hence, I always carry all valuable items with me, and I frequently visit my home to bring other stuff whenever I need something.”* (survivor, 60s, woman)

*“I stay on the second floor, and I try not to drink water in the evening. The shower is close, but the toilets are in the opposite direction, and I have to go all the way around to get there. Whenever I use the toilet during the night, I can’t sleep again.”* (survivor, 60s, woman)

*“The dining area is outside, and it is a temporary tent instead of a solid building or a room. When it is windy, rainy, or cold outside, it is cumbersome to move back and forth to eat.”* (survivor, 50s, man)

*“We do not have a separate drying room for laundry on the second floor. We hang socks and towels on a chair to dry, and our underwear is often placed in tents or hung on a sink.”* (survivor, 50s, woman)

#### 3.2.3. Service Zone

The management space did not include a separate resting area for staff and volunteers, and they had to share the dining area and residential zone with survivors. This made communication between staff and volunteers difficult as survivors could overhear their conversations. Thus, room sharing between staff and survivors resulted in community and privacy issues. In the medical space, another privacy issue emerged. The designated space for medical treatment was small, and the distance between the consultation room and waiting area was too close to maintain patients’ privacy (Table 7).

Therefore, from the interviewees’ experiences mentioned above, we inferred that the resting area should be planned in a separate space and should not be shared in common. Sufficient distance was required between the treatment space and waiting space in the medical care area by preferably providing separate rooms for each function or installing partitions between spaces.

*“When taking a break, it’s very hard for us because we don’t have our own private space. The rest area in the parking space is mainly used by the disaster survivors, and hence, we can’t just go there and sit and have conversations or take rest amid them.”* (Volunteer, 50s, woman)

*“The medical treatment room is very small. When I am waiting to receive psychological counseling, I am often embarrassed and uncomfortable because I can hear the other people’s counseling conversation, and I become worried that other people would hear my story as well.”* (survivor, 50s, woman)

#### 3.2.4. Special Needs Zone

Space for pregnant/breastfeeding women and pet areas were not included in the space plan for the shelter in Pohang. Families with infants and toddlers did not have any place to nurse babies and hence, had to return to their damaged house periodically to feed the babies. People with pets also went back to their houses to care for pets despite the danger of secondary disaster risks such as aftershocks, collapse, fire, etc. (Table 8). This showed the urgent need to provide spaces for special needs in the shelter and can be interpreted that the space plan should include areas for women to nurse their babies and pet so that infants, women, and pets can stay comfortably in the temporary shelter.

*“Because many people live together, there are hygiene issues and we are all anxious about it. Women who have babies cannot stay in the shelter because of the sanitation problem. In the beginning, there was a two-year-old baby in the shelter, but the mom and the baby left here soon.”* (survivor, 70s, woman)

*“I have dogs that have lived with me for 10 years, and the shelter does not provide a room for pets. So, I have no choice but to come home regularly and take care of them.”* (survivor, 50s, woman)

#### 3.2.5. Analysis

The following Table 9 is a comprehensive analysis matrix of habitability problems examined in the temporary shelter by zone. Most problems were pointed out in residential zones, especially in terms of comfort. In the entry zone and special needs zone, various aspects of habitability appeared relatively evenly, and the service zone lacked comfort.

## 4. Discussion

Examining the temporary shelter’s spatial problems from the perspective of habitability revealed that each zone needed improvement in the same or different categories when compared to other zones.

In the entry zone, all four aspects (safety, health, sociality, comfort) of habitability were evenly mentioned. This implied that the entry zone was not limited to a specific factor, as the entry zone was the starting point of the building’s access, crowd control, and shelter experience, so the overall aspect should be considered. In particular, “sanitation” and “area” were evaluated as relatively important elements in the entranceway. This was a place used by many people at the same time and frequently, so it required sufficient area and an effective width and supports the importance of sanitation management. The entrance door frequently used by all should open wide and the space around the door should be left free and uncrowded to support different activities such as waiting, passing, asking for help, applying for registration forms, etc.

In the residential zone, comprising personal area and public area, problems were identified in the categories of “protection/prevention” (safety), “sanitation” (health), “vulnerable people/accessibility” (sociality), “area/privacy/indoor environment quality” (comfort). Among them, it was found many comfort-related problems existed such as “area”, “privacy”, and “indoor environmental quality”. Moreover, it was emphasized that improvement was necessary for hygiene management. In the end, this fact was summarized as a problem for the qualitative management of the area and indoor environment. If the space provided to individuals is not sufficient, privacy issues will follow. If the basic indoor environment such as noise, air, light, and heat is not properly managed, an unsanitary environment is created. Therefore, space where the survivors spend most of their time, whether personal or public, should be provided with a sufficient area per individual, and the quality management of the indoor environment was closely related to promoting habitability and wellness.

In personal living spaces, the biggest problem was uniform accommodations without considering the family composition of survivors. Failure to consider sex, disability, number of family members, age, etc., eventually leads to invasion of privacy, and the provision of insufficient space also caused a great inconvenience in long-term living. This uniform space plan and minimum area standard eventually resulted in poor indoor living quality and negatively affected survivors’ physical health and psychological stability due to privacy violations.

Therefore, for personal living spaces, it is essential that (1) the area and arrangement of individual accommodations are first planned, and (2) the characteristics of members living in the accommodation must be carefully grasped in advance. Furthermore, it is legally necessary to establish a generous standard that takes into account psychological stability, without providing a minimum physical standard when arranging private accommodations or living space.

In public spaces, it was observed that the biggest problem was sharing multiple functions in one space without separating the space for each function or planning independently. Space sharing infringed on privacy between users. Therefore, when planning a public space, it is desirable to divide and provide space for each function. Besides, it is important to consider the diversity of various users. In the shelters in Pohang, there was a large shortage of dedicated spaces for managers and volunteers, and most of the space plans focused on survivors. Since this has an important relationship with the building and providing a better quality of environment for the survivors, it is necessary to consider the needs of all shelter users such as children, older adults, the disabled, managers, and volunteers.

Service zone problems were mainly seen in the categories of “area” and “privacy” in the aspect of comfort. It was identified that these problems were mainly caused by the fact that a dedicated space for managers and volunteers other than survivors was not prepared. Because a separate rest area was not provided, a privacy invasion occurred while taking a break in the corridor or using the same space as survivors. Given this fact, to improve the habitability of the service zone, it is necessary to ensure space is provided for all shelter users. Additionally, it may increase the satisfaction of shelter users and improve wellness by planning separate spaces by users or functions rather than sharing the same space for multiple purposes.

Last, the special needs zone was less habitable in the categories of “protection” (safety) and “area” (comfort). These problems were caused by the fact that hygiene, safety, and privacy were not secured because separate, independent spaces for infants, pregnant women, and nursing mothers were not provided. In the end, this problem was understood as the fact that dedicated spaces for children, pregnant women, and companion animals were not sufficiently provided. If a dedicated space was not planned, hygiene management may not be carried out thoroughly, and privacy may be infringed, making it difficult to obtain survivors’ overall welfare. Therefore, it is necessary to plan separate spaces for survivors with special needs such as children, pregnant women, and companion animals.

In summary, highlights from the findings are as follows:

- Habitability should take into account all four aspects of safety, health, sociality, and comfort, and it is essential to achieve human wellness even in the planning of a temporary disaster shelter;
- Since the entry zone is a place where a large number of users move frequently, it is necessary to secure a sufficient area and thoroughly manage hygiene;
- The residential zone is key to providing a generous space by adjusting the area provided per user above the legal minimum standard, and to thoroughly manage indoor environmental factors such as light, noise, humidity, and ventilation. In particular, in a personal living space, a plan for private accommodation that considers family members is required, and in a public space, it is necessary to separate spaces for each function;
- For service zones and special need zones, it is important to separate spaces for each function and user.

These highlights provide important insights, including:

1. Secure enough space (secure space beyond the legal minimum standards);
2. Provide sanitation and indoor environment quality management;
3. Separate spaces by function and user.

These insights are deeply related to securing the physical health of shelter users, such as survivors, managers, and volunteers, and obtaining psychological stability and privacy. They are also related to the quality and satisfaction of human life.

Since the results of the above study were conducted for one temporary housing facility in Pohang, South Korea, it is difficult to generalize the results from the temporary disaster shelter’s spatial plan. It will be necessary to add temporary shelter cases located in South Korea and expand the disaster types to floods, fires, and infectious diseases in addition to earthquakes. Furthermore, the follow-up task is to gradually increase the number of interviewees to accumulate more reliable data. These follow-up studies will help inform shelter design so that survivors acquire and maintain physical and psychological health without continuing to lose their dignity in preparation for increasing disasters.

## 5. Conclusions

The problems within the temporary shelter set up in an indoor gymnasium in Pohang revealed the need to improve the space planning and operation in terms of safety, health, sociality, and comfort to better support victims’ needs. The following are some of the recommendations based on the main research results.

First, to better support the survivors’ shelter stay, daily living activities must be performed with ease. Therefore, in preparation for various disaster situations, rather than simply switching the gym to a temporary shelter, it is necessary to review the designation and use of various types of temporary shelters carefully, such as mobile homes, welfare facilities, and lodging facilities in addition to public facilities such as gymnasiums and town halls.

Second, most of the disaster survivors staying in the temporary shelter in Pohang were older adults, and accordingly, problems such as reduced accessibility due to lack of parking spaces and frequent use of stairs to access the shelter were observed. There was also insufficient consideration for the disabled. When planning a temporary shelter, facilities and space plans for older adults and the disabled must meet the necessary standards with barrier-free access.

Third, the temporary shelter in Pohang was originally a gymnasium that was prepared for a temporary stay of one month. However, from the second field study, it was confirmed that the survivors stayed in the shelter for more than 4 months on average. Therefore, in the future, various types of disaster shelters should be developed considering the possible evacuation period, such as an emergency shelter for 2–3 days or a temporary shelter capable of supporting daily activities for a long evacuation period.

Fourth, in terms of habitability, the most important thing when planning a temporary shelter is to provide sufficient space by demarcating appropriate areas and independent spaces for each function. When spaces are shared for multiple functions, secondary problems such as accessibility, privacy, sanitation, indoor quality, and lack of protection may arise. Therefore, priority should be given to necessary functions and secure independent areas for each accordingly. Additionally, the composition and arrangement of space should consider different people’s needs based on sex, age, and user type (staff/volunteers/survivors). By ensuring concepts of habitability such as safety, health, sociality, and comfort, the temporary shelter can provide survivors with both physical health and psychological stability during a disaster.

The study was based on a case in South Korea in which a gymnasium was converted into a temporary shelter. This could be helpful in exploring the different types of shelters in the world and collecting related data. In the light of previous studies, the result of the lack of detailed and specific guidelines regarding habitability stays on the same line with previous studies. However, it can be distinguished that the research process was focused on interacting with victims, staff, and volunteers on-site instead of analyzing the literature. It is difficult to generalize the guidelines of shelters as they all have different issues by country, region, and type of disaster. Thus, examining several cases and accumulating related issues can be meaningful for the long-term development of shelter planning.

## Figures and Tables

**Table 1:** Critical concepts of habitability in temporary shelters for disaster survivors.

Main Concept	Sub-Concept	Mark	Description
Safety	Protection	Sa1	- Protection from additional disasters - Protection from bad weather conditions and potential danger - Provision of a storage system for personal items
	Prevention	Sa2	- (Emergency) lighting system for movement - Firefighting equipment considering the emergency type - Evacuation routes and exits for additional disasters - Installation of information boards on the evacuation route
Health	Sanitation	He1	- Drinking water facilities - Garbage disposal space and living area - Sanitary facilities such as toilets and showers - (Portable) sanitary facility considering the disabled and sex - Hand washing table for hygiene management - Laundry and drying space
	Medical support	He2	- Medical support space - Psychological counseling space
Sociality	Vulnerable people	So1	- Privacy for breastfeeding and childcare space - Privacy for the children’s space - Convenient movement - Simplified lifts and small ramps for wheelchairs and strollers - Information signs considering foreigners and the hearing impaired - Accessible toilets and living areas for the vulnerable - Evacuation space for companion animals
	Accessibility	So2	- Distance and travel time from the disaster area - Parking and drop-off areas for survivors and managers - Access to community facilities from the temporary shelter - Move flow for wheelchair users - Signs for temporary shelter locations and routes
	Community	So3	- Community space for watching TV, exchanging information, and eating - Communication devices such as bulletin boards and broadcasting facilities that deliver relief information
Comfort	Area	Co1	- (Minimum) area for each person considering relief supplies and disability - (3.3 m^2^ per person by the law) - Community space - Staff managers area - Installation of portable sanitation facilities and relief tents
	Privacy	Co2	- Space for individual or family unit - Configurable dividers and tents - Separate changing room for men/women
	Indoor quality	Co3	- Proper cooling and heating system - Ventilation to maintain good air quality - Secure daylights - Proper humidity management

**Table 2:** Classification of temporary shelter zones.

Main Zone	Sub-Area
Entry zone	Parking area/entrance/registration area
Residential zone	Living (sleeping) area/sanitary area/laundry and dry area/meal and prep area/rest area (lounge)
Service zone	Staff area/medical (isolation) area
Special needs zone	Childcare area/nursing area/pet area

**Table 3:** Semi-structured questionnaire framework.

Main Zone	Sub-Area	Question
Entry zone	Parking area	(1) Are parking areas planned and identifiable? (2) Is it easy to access the shelter building from the parking space? (3) Is there a parking space for emergency vehicles and cars for vulnerable people to get on and off near the main entrance?
	Entrance	(1) Are protruding obstacles removed and the area sufficiently secured? (2) Can the door be opened easily from the inside? (3) Is the entrance planned to be easily identifiable? (4) Is it wheelchair accessible?
	Registration area	(1) Is it located near the entrance? (2) Is there enough space reserved for privacy protection?
Residential zone	Living (sleeping) area	(1) Were various personnel compositions considered? (2) Has the minimum area per person been secured? (3) Are temperature and lighting properly adjustable? (4) Is natural light secured? (5) Is a storage space for personal items provided?
	Sanitary area	(1) Are toilets and showers sufficiently secured in consideration of the number of people, sex, and vulnerable people? (2) Regarding privacy, the toilets and showers are separated by sex, and shower curtains are installed?
	Laundry and dry area	(1) Are there separate laundry and drying spaces?
	Meal and prep area	(1) Is it located in a separate place from the living space? (2) Is a hygienic environment secured? (3) Are there preparation spaces for meal supply and distribution?
	Rest area (lounge)	(1) Is it located in a place with low noise?
Service zone	Staff area	(1) Are entrances and movement flows secured separately for managers and volunteers?
	Medical area	(1) Is it separated from the living space for privacy protection? (2) Has the minimum area per person been secured?
Special needs zone	Childcare area	(1) Can children access the space? (2) Are separate spaces provided for children to play safely?
	Nursing area	(1) Are toilets separated by sex? (2) Are changing rooms, nursing spaces, and childcare spaces provided?
	Pet area	(1) Have other people staying in the shelter been considered? (2) Has the area, isolation distance, and drainage facilities been secured in consideration of the type and size of companion animals?

**Table 4:** Problems identified in the entry zone.

**Category**	**Mark**	**Main Problems**
Parking area (En1)	e-1	Lack of parking space for survivors because of snack bars, dining booths, and laundry vehicles in the parking space
		Keywords: lack of parking space for survivors
	e-2	Garbage disposal in the parking lot increasing congestion, causing an odor, and thus, an unsanitary environment
		Keywords: garbage disposal and odor, congestion
	e-3	No separate sidewalk provided in the parking space exposing the survivors to a potential accident
		Keywords: no separate sidewalk, potential car accident
Entrance (En2)	e-4	A bottleneck at the main entrance during the evacuation after the aftershock; no signage provided for accessing the emergency exit
		Keywords: bottleneck at the main entrance, no signage for accessing emergency exit
	e-5	Contamination of entrance door handles and surfaces due to the frequent use resulting in an unsanitary environment
		Keywords: door contamination
	e-6	Narrow entrance causing congestion when many people use it at the same time
		Keywords: narrow entrance width, congestion
Registration area (En3)	e-7	Shared space arrangement with management space and a cell phone charging station in the crowded entrance lobby; no protection of privacy during the registration process in this open space
		Keywords: space sharing, privacy

**Table 5:** Problems identified in the residential zone (1).

Category	Mark	Main Problems
Living/Sleeping area (Re1)	r-1	Lack of storage space for personal items; storage space far from the tents exposes theft risk
		Keywords: lack of storage for personal items, potential theft risk
	r-2	Narrow space between tents made people uncomfortable
		Keywords: a narrow passage
	r-3	Potential exposure to a secondary disaster when survivors periodically return to damaged houses to bring stuff owing to lack of storage space for necessities (clothes, toiletries, etc.)
		Keywords: secondary disaster exposure, lack of storage for personal items
	r-4	Uniform tent size with no consideration for family size, personal characteristics (sex, height, etc.), and number of personal items
		Keywords: same-sized tent with no flexibility
	r-5	Sleep disturbance caused by excessive noise (walking, coughing, etc.) heard across narrow gaps between tents
		Keywords: narrow gaps between tents, excessive noise
	r-6	Bedding becoming wet or moldy due to condensation on the floor of the tent
		Keywords: wet/moldy bedding, condensation on the tent floor
	r-7	Open structure in the center causing dust generated on the 1st floor to rise and affect people’s health staying on the 2nd floor; stinging sensation in the eyes and neck
		Keywords: ventilation and dust care, open space
	r-8	Survivors staying on the 2nd floor have poor accessibility to mobile phone charging stations and laundry vehicles on the 1st floor and have to use stairs
		Keywords: stair use for facility access
	r-9	Tent arrangement with no consideration for sex and family units, resulting in privacy issues
		Keywords: no optional tent sizes and structures, privacy
	r-10	Low humidity due to excessive heating during winter causing health problems such as colds and rhinitis
		Keywords: excessive heating, low humidity, health issues
	r-11	Residential space on the second floor adjacent to a window leading to exposure to cold weather
		Keywords: living area at the window side, exposure to cold weather

**Table 6:** Habitability problems identified in the residential zone (2).

Category	Mark	Main Problems
Sanitary area (Re2)	r-12	Sanitary space arrangement without considering sex and privacy
		Keywords: space arrangement without considering sex, privacy
	r-13	Discomfort among older adults as the toilets were far from their living space
		Keywords: long distance and poor access to toilets, older adults
	r-14	Privacy infringement due to absence of shower compartments
		Keywords: no shower compartment provided, privacy
	r-15	Insufficient hot water supply considering the number of survivors, causing people to use external facilities
		Keywords: insufficient hot water supply
Laundry/Drying area (Re3)	r-16	Lack of drying space, personal laundry exposed to others if dried outside the tent
		Keywords: no laundry drying space, privacy
	r-17	Drying personal laundry inside the tent causes high humidity, odor, respiratory infections, etc.
		Keywords: underwear drying inside a tent, poor indoor quality
	r-18	Personal laundry, such as underwear, is washed in a public washstand, causing exposure to an unsanitary environment
		Keywords: no place for hand wash laundry, sanitary environment
Dining/Meal-prep. Area (Re4)	r-19	Dining area in the parking lot exposed survivors to extreme weather conditions (rain, snow, strong wind)
		Keywords: dining area location, no protection for people from extreme weather conditions
	r-20	Dining spaces reduced to one after a prolonged shelter stay, lack of dining space for survivors
		Keywords: insufficient dining area
	r-21	Problems with freezing food materials stored in the outdoor meal preparation area
		Keywords: no separate refrigerator for public use
Rest area (Re5)	r-22	No separate resting area provided indoors resulted in people sitting on the floor, occupying empty spaces, talking loudly, and exposing private conversations and information
		Keywords: no separate resting area provided
	r-23	Space sharing in the dining area caused congestion
		Keywords: space sharing in the dining area, congestion

**Table 7:** Problems identified in the service zone.

Category	Mark	Main Problems
Staff area (Se1)	s-1	No separate resting area for managers and volunteers; difficult to protect privacy due to space sharing
		Keywords: no separate resting area for staffs/volunteers, privacy
Medical area (Se2)	s-2	Privacy infringement in psychological counseling space as it is separated by a narrow space from the waiting area; not enough space in the medical treatment area
		Keywords: a small area for medical care, privacy issue due to close spacing

**Table 8:** Problems identified in the special needs zone.

**Category**	**Mark**	**Main Problems**
Nursing area (Sp1)	sn-1	No room provided for nursing babies in the shelter and families with infants and toddlers returned to their damaged houses to nurse babies, thereby risking exposure to a secondary disaster
		Keywords: no separate space, secondary disaster exposure
Pet area (Sp2)	sn-2	No space plan for pets in/around the shelter; people with pets return to their damaged houses to take care of pets with a risk of secondary disaster exposure
		Keywords: no separate space, secondary disaster exposure

**Table 9:** Analysis matrix.

	Zone	Entry Zone			Residential Zone					Service Zone		Special Needs Zone	
Category		En1	En2	En3	Re1	Re2	Re3	Re4	Re5	Se1	Se2	Sp1	Sp2
Sa1		e-3	-	-	r-1 r-3	-	r-16	r-19	-	-	-	sn-1	sn-2
Sa2		-	e-4	-	r-2	-	-	-	-	-	-	-	-
He1		e-2	e-5	-	r-6 r-7	r-15	r-16 r-17 r-18	-	-	-	-	sn-1	-
He2		-	-	-	-	-	-	-	-	-	s-2	-	-
So1		-	-	-	r-4 r-8	r-13	-	-	-	-	-	sn-1	sn-2
So2		e-1	-	-	r-2 r-8	r-13	-	r-19	-	-	-	-	-
So3		-	-	-	-	-	-	-	-	-	-	-	-
Co1		-	e-6	e-7	r-1 r-2 r-3	-	r-16 r-17	r-20 r-20	r-22 r-23	s-1	s-2	sn-1	sn-2
Co2		-	-	e-7	r-2 r-4 r-5 r-9	r-12 r-14	r-16	-	r-22	s-1	s-2	-	-
Co3		-	-	-	r-5 r-6 r-7 r-10 r-11	-	r-17	-	-	-	-	-	-
